# Quantification in shooting precision for preferred and non-preferred foot in college soccer players using the 95% equal confidence ellipse

**DOI:** 10.3389/fspor.2024.1434096

**Published:** 2024-09-13

**Authors:** Yusuke Shimotashiro, Masahiro Shinya

**Affiliations:** Graduate School of Humanities and Social Sciences, Hiroshima University, Hiroshima, Japan

**Keywords:** variability, accuracy, football, kick, motor control, bivariate normal distribution

## Abstract

Shooting precision is a fundamental characteristic in soccer, yet the probabilistic structure and magnitude of precision in soccer shooting remain quantitatively unexplored. This study aimed to quantify shooting precision using measures derived from the bivariate normal distribution for both preferred and non-preferred feet. Sixteen right-footed collegiate soccer players participated by performing instep kicks aiming at targets which are placed close to the left and right top corners of the soccer goal. We used bivariate normal distribution modeled the ball positions, revealing an ellipsoidal distribution, and the area of the 95% confidence ellipses served as an index of precision. Repeated measures ANOVAs revealed a significant main effect of the kicking foot. For shots aimed at the same side as the kicking foot, the area of the 95% confidence ellipse was 6.17 ± 1.93 m^2^ (mean ± SD) for the preferred foot and 10.22 ± 3.53 m^2^ for the non-preferred foot. Similar results were observed for shots aimed at the opposite side of the kicking foot. These quantitative findings hold promise for advancing soccer research and enhancing practical applications in soccer skill assessment.

## 1 Introduction

In soccer, majority of goals are scored by foot, with more than 80% of goals at the 2006 and 2010 World Cup matches achieved this way. Notably, 85% of these goals were scored using the right foot (1, 2). Almeida et al. (3) analyzed success rate of 536 penalty kick attempts during a five-year UEFA-sponsored match, focusing on the influence of the kicking foot and shooting direction. Their findings revealed that right-footed players had a higher success rate for penalty kicks aimed at the right side of the goal (from the kicker's perspective) compared to those aimed at the left side, with a similar pattern observed for left-footed players (3). These real-game observations point to potential differences in physical and/or motor control abilities influenced by the kicking foot and shooting direction.

Asymmetries in in-game preference and performance between the preferred and non-preferred legs have been reported. Previous studies have documented differences in the frequency of usage between the preferred and non-preferred feet. Carey et al. (4) found that the preferred leg was used more frequently for first touches and dribbling in the 1998 FIFA World Cup. Similarly, Marcori et al. (5) reported a higher frequency of shooting with the preferred foot in European league matches. This preference extends to amateur players as well (6, 7). While the asymmetry in preference is observed in both professional and amateur players, the impact on play quality, such as pass success rates, differ between these groups. Professional players show no significant difference in the quality of plays between their preferred and non-preferred feet (4, 5), whereas a marked difference is evident in amateur players (6–8). These findings suggest that asymmetry in shooting accuracy could provide valuable insights for assessing player performance levels.

The influence of the shooting direction on kick accuracy was explored by Nagasawa et al. (8). They placed targets in the corner of a goal and instructed participants to shoot from a penalty kick (PK) distance aiming at these targets. As an assessment of shooting accuracy, they employed a research paradigm in which they count scores depending on results of shots: A shot hitting the target earned 2 points, a shot hitting the target frame received 1 point, and all other shots scored 0 points. Using this method, they reported that shots executed with the dominant foot achieved higher scores than those with the non-dominant foot. Additionally, straight shot (a shot aiming at the right target with the right foot, and a shot aiming at the left target with the left foot) yielded higher scores compared to cross shots (a shot aiming at the target on the opposite side of the kicking foot).

**Figure 1:**
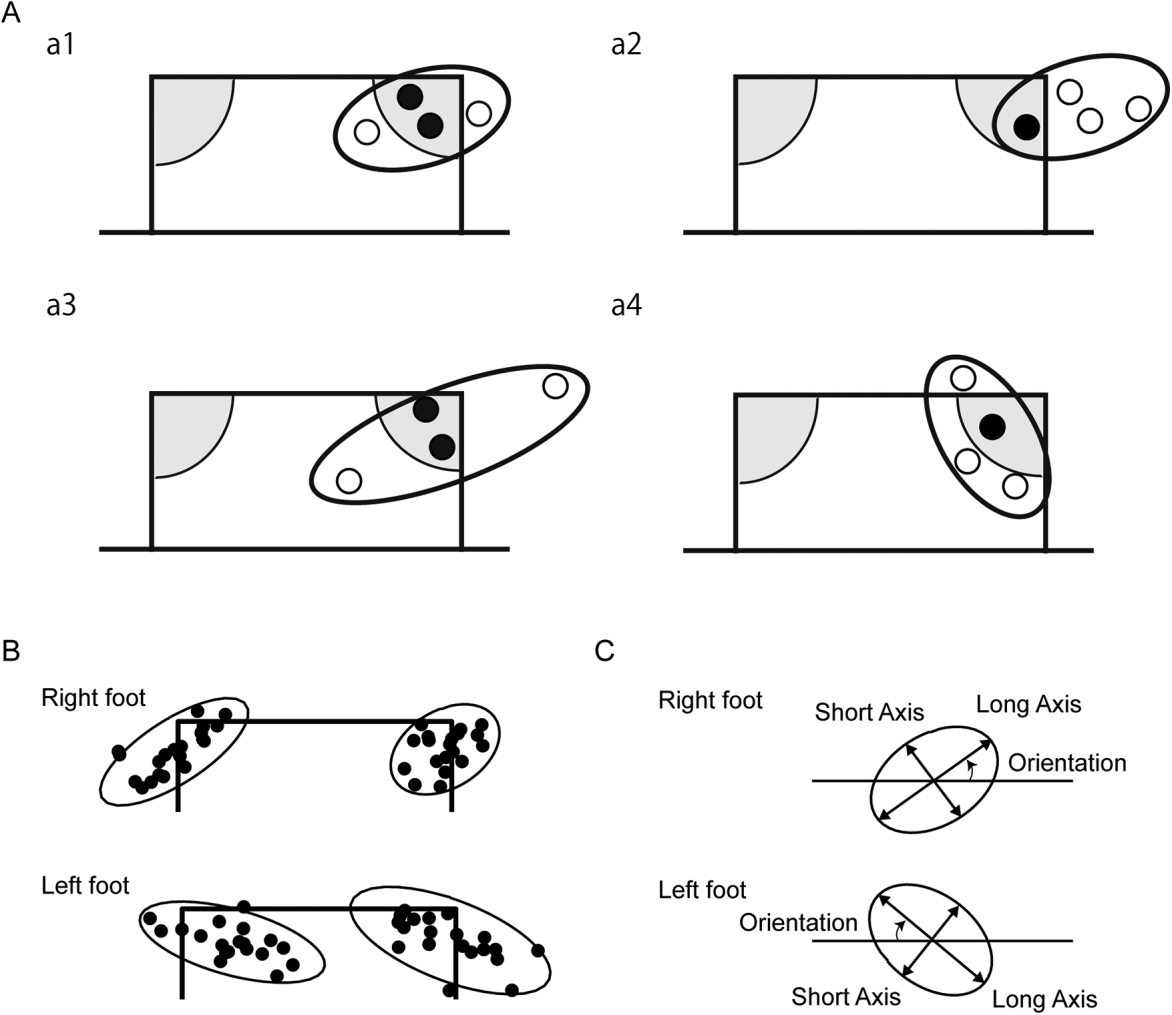
Quantification of shooting precision using the 95% equal confidence ellipses. **(A)** Comparison between a conventional point-based method and the analytical method using 95% equal confidence ellipse. For a point-based method, discrete points are assigned for specific areas in the goal: grey areas at the top corners [e.g., (8)]. In the present study, we quantified the variability in the ball position using the 95% equal confidence ellipse. Schematic examples of ball position of 4 shots are illustrated in sub panels a1–a4. Black circles indicate balls that hit the target, while the white circles indicate balls that missed the target based on the conventional method. **(B)** Ball positions of shots of one participant and 95% equal confidence ellipses for each condition were illustrated. **(C)** Based on the 95% equal confidence ellipse, the long axis, short axis, and the area were calculated as the indices of the size of the shooting precision. The orientation of the ellipse was calculated as the angle of the long axis from the horizontal line. Note that the sign of the orientation was flipped to compare between the right and left kicking foot conditions.

Traditional methods for evaluating accuracy in sports performance typically rely on enumerating successful target hits [Nagasawa et al. (8) in soccer and Wagner et al. (9) in handball]. In other research, the goal was divided into subareas and different scores were assigned to each for evaluating soccer shots (10, 11). However, these conventional scoring paradigms conflate the concepts of accuracy (the systematic error between the target and the centroid of ball placements) and precision (the dispersion of ball placements). Consequently, disparate score evaluations may arise even when the underlying variability remains constant (Figure 1A, a1 vs. a2). Furthermore, these methods fail to account for the magnitude of deviation within zero-score regions, potentially obscuring significant differences in precision (Figure 1A, a1 vs. a3). Lastly, the orientation of the ball distribution, which is the essential feature of two-dimensional variable such as ball position, is also neglected (Figure 1A, a1 vs. a4). Previous research has highlighted the importance of the orientation of the endpoint distribution as a fundamental attribute of human motor control, which depends on the involved limb or movement direction (12, 13). Therefore, our methodology aimed to quantify not only the magnitude but also the orientation of variability in shot placements.

An analytical approach involves calculating absolute error and variable error. For instance, variable error, which is often represented by the standard deviation, has been separately calculated for horizontal and vertical coordinates in sports such as cricket (14) and baseball pitching (15). However, as horizontal and vertical data may be correlated and ball positions might form an ellipsoidal shape, multidimensional analysis is warranted. Recent studies by Shinya et al. (13) and Hunter et al. (16) used the bivariate normal distribution to measure two-dimensional precision in sports performance. They fit a 95% equal confidence ellipse to the distribution of ball positions and used the area, long axis, and short axis of the ellipse as outcome measures for assessing precision (Figure 1C).

Ball velocity should be assessed when quantifying precision. The speed-accuracy tradeoff is a well-established principle in motor control (17, 18), which posits that one can enhance accuracy in motor tasks by reducing movement velocity. Rakojević et al. (19) have demonstrated that this trade-off also applies to soccer kicking. We aimed to assess whether the relative ball velocity to each individual's maximum velocity remains consistent across kick conditions, thereby confirming that any differences in precision were not confounded by changes in speed-accuracy strategy. Additionally, we sought to examine whether ball velocity could be a determinant of ball position (20). Gordon et al. (12) argued that the elliptical distribution of arm reaching endpoints reflects independent control mechanisms for direction and amplitude of motion. Extending this concept to soccer, ball velocity may influence the variation of the ball along the long or short axes of the 95% equal confidence ellipse. To assess whether the ball velocity determines the ball position for each shot, we calculated correlation coefficients between ball velocity and the ball's position along the long and short of the 95% confidence ellipse for each tested condition.

The aim of this study was to examine the impact of kicking foot and shooting direction on precision in soccer shooting. To measure shot precision, we analyzed ball positions using the bivariate normal distribution. The initial inquiry was related to the probabilistic structure of the ball positions for soccer shooting: whether the distribution of the ball position in soccer shooting was circular or ellipsoidal. We posed two hypotheses related to precision. The first hypothesis was that shooting precision would be superior for the preferred limb compared to the non-preferred limb. The second hypothesis was that shot precision would be greater for the straight direction compared to the cross direction. This conjecture would be substantiated by observing a reduced area, as well as shorter long and short diameter of the confidence ellipse for shot by the preferred limb aimed at a target set at the straight direction.

## 2 Materials and methods

### 2.1 Sample size determination

To assess our hypotheses, we planned to employ two-way repeated measures ANOVAs (rmANOVA) with within-subject factors of kicking foot (preferred and non-preferred) and shooting direction (straight and cross). A power analysis for the repeated measures ANOVA (rmANOVA) was performed using G*Power version 3.1.9.7. For the expected effect size, a large effect [*η*^2^ = 0.40 (21)] was used in the power analysis. The choice was based on a previously reported main effect of kicking foot on the variability of ball position which was *η*^2^ = 0.51 (22), and main effect of kicking direction on variability of ball position which was *η*^2^ = 0.46 (23). The other parameters used in the power analysis were as follows: *α* = 0.05, 1 − *β* = 0.8, number of groups = 1, number of measurements = 2, correlation among repeated measures = 0.5. The suggested sample size was 15. Considering the need to counterbalance the order of conditions tested, we planned to recruit 16 participants for this study. We also confirmed that our research plan of recording 20 trials from 16 participants was capable of detecting a significant difference with 83% statistical power, given a 1.2 times within-subject difference in the standard deviation (24).

### 2.2 Research ethics and participant recruitment

This study was approved by the Ethics Committee of the Faculty of Integrated Arts and Sciences, Hiroshima University (Approval number: 03-47). The inclusion criteria for participation in the study were age 18 to 30, right-footed, and had at least six years of soccer experience. The preferred foot was defined as the kicking foot used predominantly during matches. Participants with a history of lower limb injuries affecting soccer play were excluded from the study. Recruitment of participants was conducted through printed flyers and online advertisements. As a result, 16 participants (13 males and 3 females) with a mean age of 20.6 ± 2.1 years, a mean height of 169.5 ± 5.63 cm, and an average soccer experience of 9.75 ± 2.59 years participated in the study. The participants’ soccer level was recreational. Written informed consent was obtained from all participants before the commencement of the experiment.

### 2.3 Experimental tasks

**Figure 2:**
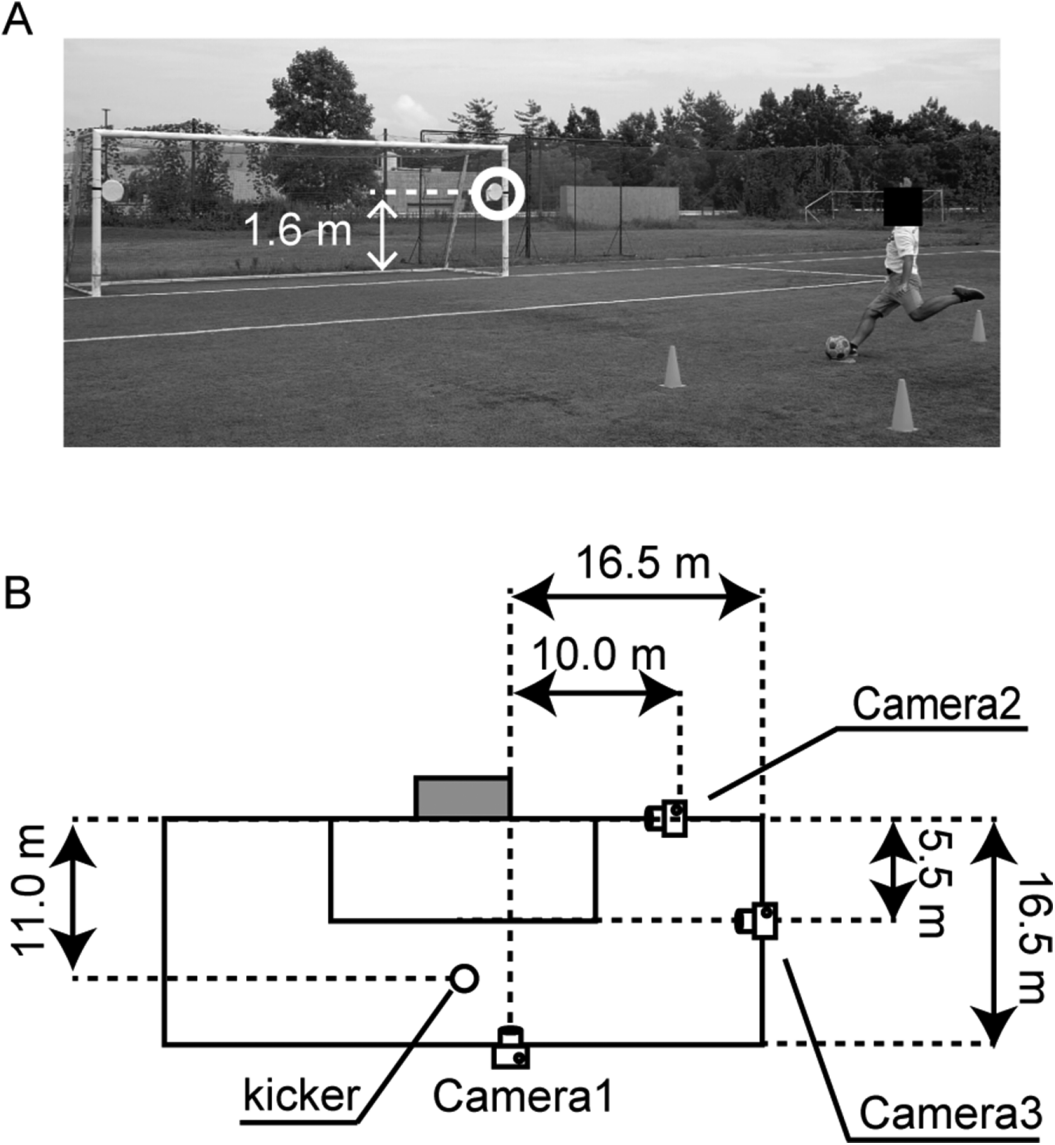
Experimental setup. **(A)** Experimental setup. A photo is shown when the kicker aimed at the target on the right side with his left foot. **(B)** Camera placement. The figure shows the camera placement when the kicker aimed at the target on the right side. In the condition of aiming at the target on the left side, the camera placement was symmetrical with the center of the goal as the axis Measurements.

An experimental task required to shoot from the penalty mark with an instep kick aimed at a circular target of 0.33 m in diameter placed inside the goalpost. The distance from the center of the goal to the kicking point was 11 meters. The target was positioned at a height of 1.6 meters, following the reference of Hunter et al. (16) (Figure 2A). A 2 m × 4 m area was set up as the participant's run-up area. The participants were instructed to shoot as fast and as accurately as possible. They were also directed to approach and kick the ball just as they would during a penalty kick in a match, maintaining a consistent run-up direction regardless of the target side. Targets were placed on both the right and left sides and shots were performed using preferred foot (right foot) and non-preferred foot (left foot).

Before the start of the experiment, participants were given a 20 min warm-up. Subsequently, maximum ball velocity measurements were taken for both the right and left feet, with two trials for each. During these measurements, no specific target was set, and participants were instructed to kick their fastest shots toward the center of the goal. After, the participants performed a total of 80 trials, 20 for each condition, using a block design. The order of conditions was counterbalanced between participants. The order of conditions was counterbalanced between participants (see Table 1 for the detail). Before the measurement of each condition, participants completed five practice trials. Finally, maximum ball velocity measurements were taken for both the right and left feet, with two trials for each.

**Table 1:** Counterbalancing the order of conditions.

1	RS	LC	RC	LS
2	RS	LC	LS	RC
3	LC	RS	RC	LS
4	LC	RS	LS	RC
5	RC	LS	RS	LC
6	RC	LS	LC	RS
7	LS	RC	RS	LC
8	LS	RC	LC	RS

Eight sequences of tested conditions were created to ensure proper counterbalancing. Two participants were assigned to each sequence. RS, straight kicks by the right foot; RC, cross kicks by the right foot; LS, straight kicks by the left foot; LC, cross kicks by the left foot.

We used three time-synchronized cameras for our measurements (Figure 2B). Time synchronization was performed by dropping the ball from the hand and timing its contact with the ground. Camera 1 (GH5, Panasonic, 3,840 × 2,160 pix, 120 fps) was set up in front of the goalpost on the side where the target was aimed. Camera 1 underwent a two-dimensional Direct Linear Transformation (DLT) calibration using the goal line and vertical line to measure the ball position when it passed the goal line (referred to as the shot position). Camera 2 (FDR-AX45, Sony, 1,920 × 1,080 pixels, 60 fps) was placed outside the goalpost on the side where the target was, and it measured the timing of the ball crossing the goal line. Camera 3 (iPhoneXS, Apple, 1,920 × 1,080 pixels, 60 fps) was positioned parallel to the touchline, covering the entire shooting experiment, including the kicker and the goal. Camera 3's footage was used to visually determine the timing when the ball started to move during the shot. The ball velocity during the shot was calculated as (*t*_1_ − *t*_2_)/*d*, where *t*_1_ is the time of the ball crossing the goal line, *t*_2_ is the time the ball started to move, and d is distance from penalty mark to ball position.

## 3 Analysis

Trials in which the ball made contact with the ground before reaching the goal line were excluded from the analysis. As a simple measure of kick accuracy, we counted the number of hitting and missing the target for each condition. Maximum ball velocity was calculated from four trials, two trials of maximum ball velocity before and two trials of maximum ball velocity after measuring ball variability. The variability in the ball positions was analyzed based on the bivariate normal distribution for each subject and condition (13, 16, 25). We analyzed the long axis, the short axis, and the area of the 95% equal confidence ellipse.

The orientation of the 95% equal confidence ellipse is also the important aspect of the two-dimensional variability if the distribution has an anisotropic feature (26). To test the anisotropy of the distribution, we compared the long and short axes length using an F-test. Specifically, when the ratio was over 1.59, which corresponds to the upper limit of 95% confidence interval of *F*(19, 19), the distribution was regarded as ellipsoidal and the orientation of the long axis defined as the angle from the horizontal line was analyzed. The positive angle means that the ball positions are distributed in a kicking-foot-side-up and supporting-foot-side-down ellipse (Figures 1B,C).

It would be interesting to see what factors determine the position of the ball within the elliptical distribution. It has been reported that physical parameters of a ball such as ball velocity, launch angle, and ball spin are determinants of the final ball position (20). Although we did not measure the detailed ball parameters because the primary objective of the present study was to quantify the variability in ball position, we analyzed the relationship between the ball velocity and ball position. The ball position was transformed into a coordinate system based on the distribution of the ball position, and then Pearson's correlation coefficient between the long and short axis coordinate and the ball velocity was calculated for each subject and condition.

## 4 Statistics

To ascertain the effect of fatigue on the experiment, the difference between the maximum ball velocities performed at the beginning and end of the experiment was evaluated using a paired *t*-test. We used a two-way ANOVA with kicking foot and shooting direction as independent variables for the number of times the ball hit the target. Prior to statistical analysis, the shooting direction conditions were redefined as straight (right foot to right target or left foot to left target) and cross (right foot to left target or left foot to right target). For comparing variability measures (i.e., the area, short axis, and long axis), we first took logarithm of the variables before performing a two-way repeated measures ANOVA according to the recommendation of a previous study (24).

We used circular statistics to analyze the between-subjects mean of the orientation of the long axis of the 95% equal confidence ellipse and the 95% confidence interval as descriptive statistics for the direction of the ball position variation. We analyzed whether the orientation of the long axis differed between experimental conditions using circular statistics corresponding to a paired *t*-test. For comparisons, the sign of the orientation of the long axis was reversed for the non-preferred foot condition. To allow for multiple comparisons, the significance level was set at 0.0083 = 0.05/6, following Bonferroni's method. The speed-accuracy tradeoff is a well-established phenomenon in motor control (17, 18). In soccer specifically, it has been reported that as ball velocity increases, so does ball trajectory variability (19). Additionally, previous studies have shown that variability in reaching tasks depends on the ratio of output force to maximum force (27). Given the importance of the ratio between task velocity and maximum velocity, we calculated the ratio of ball velocity during the experimental task to the maximum ball velocity for each subject and condition. These ratios were then compared across conditions using two-way repeated measures analysis of variance. The ratios of ball velocity to maximum ball velocity during the experimental task were then compared across conditions using two-way repeated measures analysis of variance.

Pearson's product-rate correlation coefficients were transformed into z-values using the Fisher's *z*-transform. The mean and upper and lower limits of the 95% confidence interval were calculated for the *z*-values obtained. The results and figures indicate the inverse *z*-transformation to *r*-values for the mean and upper and lower bounds of the 95% confidence interval for the *z*-values. We compared *z*-values across conditions using two-way repeated measures analysis of variance. Because we observed a significant interaction between shooting direction and kicking foot, we adjusted the significance level by Bonferroni correction (*α* = 0.0083 = 0.05/6) before comparing across conditions.

We used JASP (ver. 0.16) for *t*-tests and analysis of variance. For circular statistics, the Toolbox for circular statistics in MATLAB was used (28). The significance level was set at 0.05, excluding correction for multiple comparisons. In the description of the results in this paper, descriptive statistics are expressed as mean ± standard deviation, unless otherwise noted.

## 5 Results

Forty-four kicks (preferred foot: 17, non-preferred foot: 27; 0–5 trials per participant) were excluded from the analysis based on the criterion that the ball contacted the ground before reaching the goal line. No significant differences were observed between the ball velocity performed at the beginning and end of the experiment (Table 2), which suggests a minimal influence of the fatigue on the experiment. Out of 20 attempts of kicks by the right foot, the average number of times the ball hit the target was 1.27 (0–4, min and max) times for the straight direction and it was 1.62 (0–6) times for the cross direction. None of the participants hit the target once or more when they kicked by the left foot.

**Table 2:** Maximum ball velocity.

Subject	Pre		Post	
	Right foot	Left foot	Right foot	Left foot
	Absolute velocity (m/s)	Absolute velocity (m/s)	Absolute velocity (m/s)	Absolute velocity (m/s)
1	21.8	20.7	23.6	19.4
2	20.8	17.6	21.6	18.1
3	18.3	15.3	20.3	15.7
4	21.6	17.7	23.2	19.3
5	24.2	22.3	21.6	22.0
6	23.6	21.7	20.9	20.5
7	23.2	22.2	23.6	23.0
8	23.4	21.8	24.9	23.0
9	17.9	16.7	18.9	16.7
10	18.9	19.2	19.7	19.6
11	19.9	18.6	19.6	19.3
12	27.8	25.7	26.9	25.4
13	23.6	22.6	25.7	23.2
14	23.7	15.6	23.4	20.5
15	22.8	21.7	22.6	20.4
16	24.0	22.0	26.7	24.7
Average	22.2	20.1	22.7	20.7

**Figure 3:**
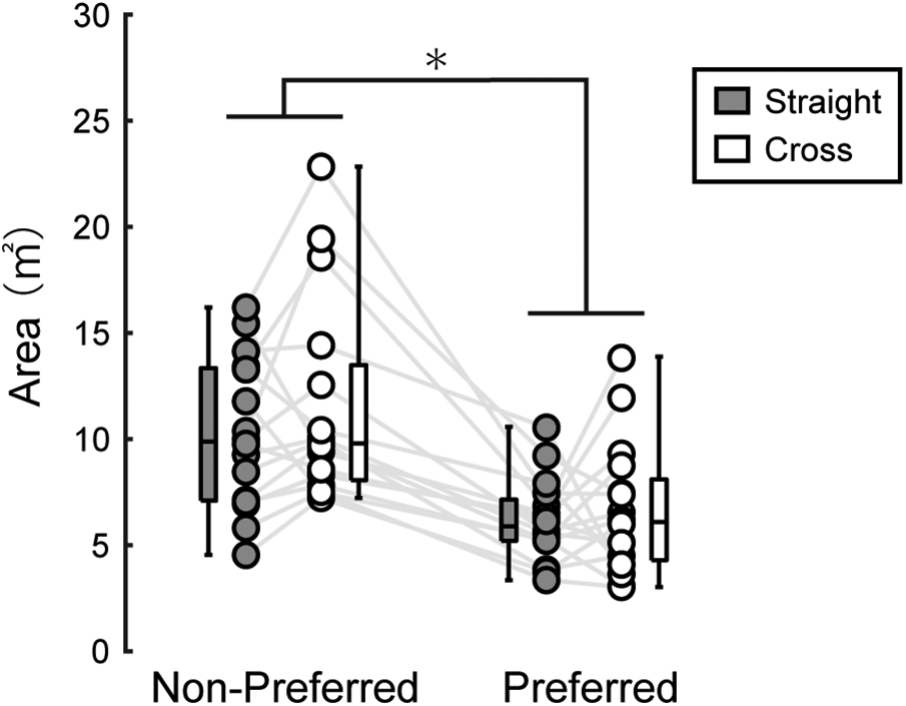
Area of the 95% confidence ellipse. Dots connected with a line represent data from the same participant. The distribution of the data is depicted using box plots, showing minimum, maximum, median, and first and third quantiles. Although statistical analyses were conducted on the logarithms of the variable, the figure presents the raw values. A significant main effect of kicking foot was identified by a two-way repeated ANOVA (* in the figure, *p* < 0.05), while the main effect of shoot direction and the interaction were not statistically significant.

The area of the 95% equal confidence ellipse was 6.17 ± 1.93 m^2^ in the straight direction and 6.62 ± 3.10 m^2^ in the cross direction for the preferred foot. For the non-preferred kick, it measured 10.22 ± 3.53 m^2^ in the straight direction and 11.50 ± 4.81 m^2^ in the cross direction (Figure 3). A two-way repeated measures ANOVA revealed a significant main effect of kicking foot [*F*(1, 15) = 57.18, *p* < 0.001, *η*^2^ = 0.55]. The main effect of shooting direction and the interaction between kicking foot and shooting direction were not statistically significant.

**Figure 4:**
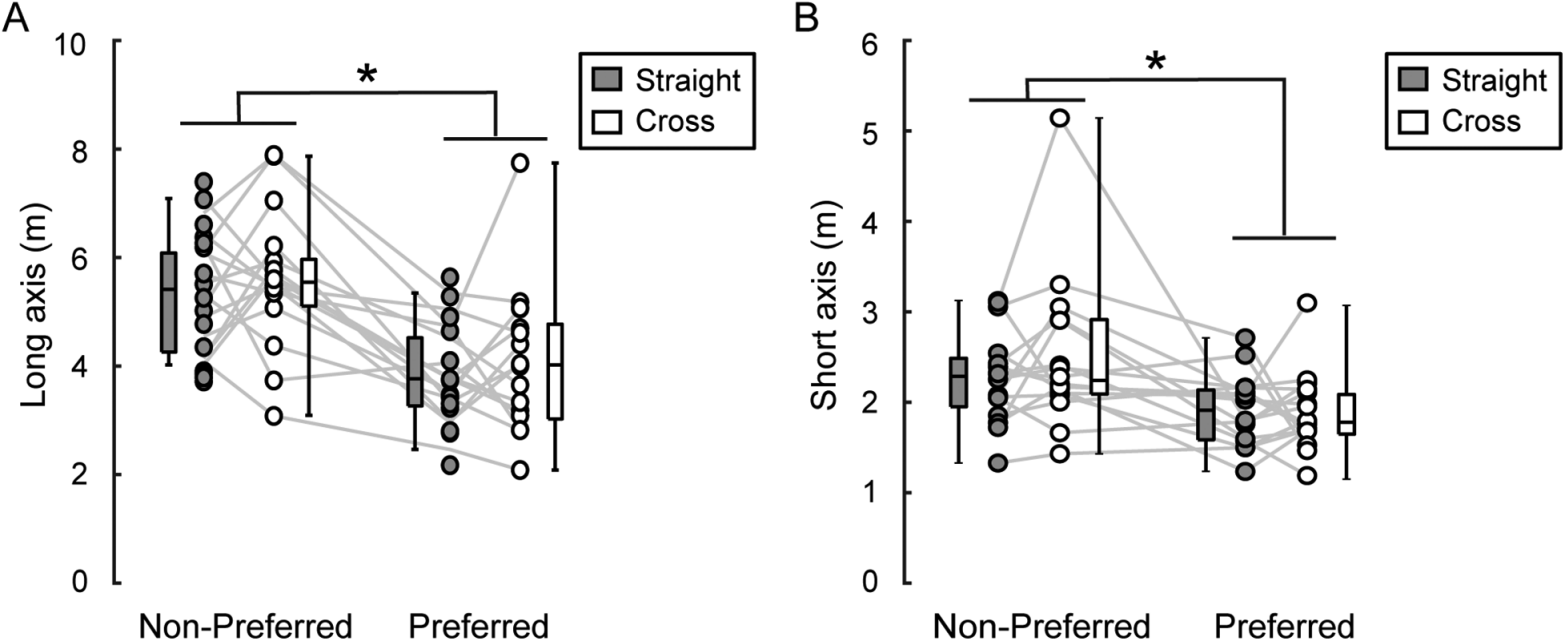
Long and short axes of 95% equal confidence ellipse. The figure displays long axis **(A)** and short axis **(B)** Dots connected with a line represent data from the same participant. The distribution of the data is depicted using box plots, showing minimum, maximum, median, and first and third quantiles. Although statistical analyses were conducted on the logarithms of the variable, the figure presents the raw values. A significant main effect of kicking foot was identified by a two-way repeated ANOVA (* in the figure, *p* < 0.05), while the main effect of shoot direction and the interaction were not statistically significant.

The long axis of the 95% equal confidence ellipse were 4.08 ± 0.81 m in the straight direction and 4.32 ± 1.34 m in the cross direction for the preferred foot. For the non-preferred foot, it was 5.60 ± 1.02 m in the straight direction and 5.83 ± 1.28 m in the cross direction (Figure 4A). A two-way repeated measures ANOVA revealed a significant main effect of kicking foot [*F*(1, 15) = 116.12, *p* < 0.001, *η*^2^ = 0.55]. The main effect of shooting direction and the interaction between kicking foot and shooting direction were not statistically significant.

The short axis of the 95% equal confidence ellipse were 1.91 ± 0.39 m in the straight direction and 1.89 ± 0.43 m in the cross direction for the preferred foot. For the non-preferred foot, it was 2.28 ± 0.51 m in the straight direction and 2.51 ± 0.86 m in the cross direction (Figure 4B). A two-way repeated measures ANOVA revealed a significant main effect of kicking foot [*F*(1, 15) = 11.21, *p* = 0.004, *η*^2^ = 0.26]. The main effect of shooting direction and the interaction between kicking foot and shooting direction were not statistically significant.

**Figure 5:**
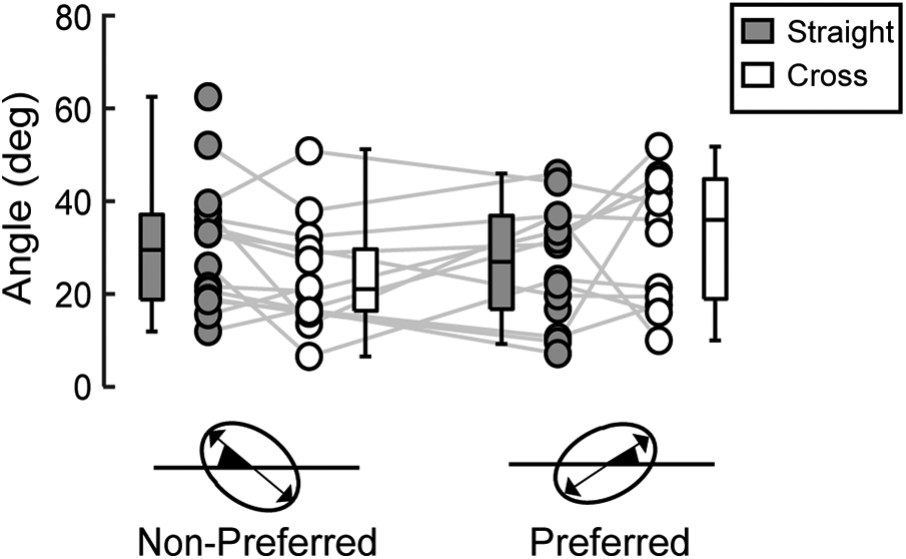
Angle of 95% equal confidence ellipse. Dots connected with a line represent data from the same participant. The distribution of the data is depicted using box plots, showing minimum, maximum, median, and first and third quantiles. No significant differences were observed between all the conditions.

Among a total of 64 distributions of ball positions (16 participants × 4 conditions), 57 were considered ellipses. A comparison of the orientation of the long axis of the 95% equal confidence ellipses calculated for these 57 distributions of ball positions using circular statistics revealed no significant differences in the orientation of the long axis between conditions (Figure 5, *p* > 0.05).

The absolute and relative ball velocities for each condition were shown in Table 3. The results of the analysis of variance with the relative ball velocity during the experimental task as the dependent variable showed that the interaction between shooting direction and kicking foot, the main effect of kicking foot and the main effect of shooting direction, were all not significant.

**Table 3:** Ball velocities during the experimental conditions and their relative values compared to each participant's maximum ball velocity.

Subject	Right foot/straight		Left foot/straight		Right foot/cross		Left foot/cross	
	Absolute velocity (m/s)	Relative velocity (%)	Absolute velocity (m/s)	Relative velocity (%)	Absolute velocity (m/s)	Relative velocity (%)	Absolute velocity (m/s)	Relative velocity (%)
1	22.0	97.0	19.1	95.4	20.6	91.0	20.8	103.8
2	19.7	92.7	15.9	89.3	19.0	89.6	16.8	94.0
3	18.8	97.3	15.2	98.5	18.8	97.5	16.4	106.0
4	21.8	97.4	16.6	89.9	21.1	94.4	18.8	101.8
5	21.9	95.6	21.1	95.2	23.2	101.1	22.1	100.0
6	21.7	97.6	20.1	95.3	21.3	95.5	19.9	94.3
7	23.5	100.4	21.0	92.9	22.3	95.4	22.9	101.5
8	24.2	100.1	21.0	93.5	23.2	96.0	21.6	96.2
9	17.4	94.3	15.0	89.5	16.4	88.8	15.1	90.2
10	19.2	99.8	17.4	90.0	19.1	99.0	16.9	87.2
11	17.5	88.9	17.2	91.0	18.0	91.2	17.6	93.1
12	24.6	90.0	22.7	88.9	25.2	92.0	22.2	87.1
13	24.1	97.8	20.8	90.8	24.2	98.1	21.2	92.9
14	22.1	94.1	15.2	84.1	21.3	90.6	18.0	99.5
15	21.9	96.6	19.9	94.5	22.3	98.4	20.5	97.2
16	24.4	96.2	21.6	92.4	23.1	91.2	20.8	89.1
Average	21.6	96.0	18.7	91.9	21.2	94.4	19.5	95.9

**Figure 6:**
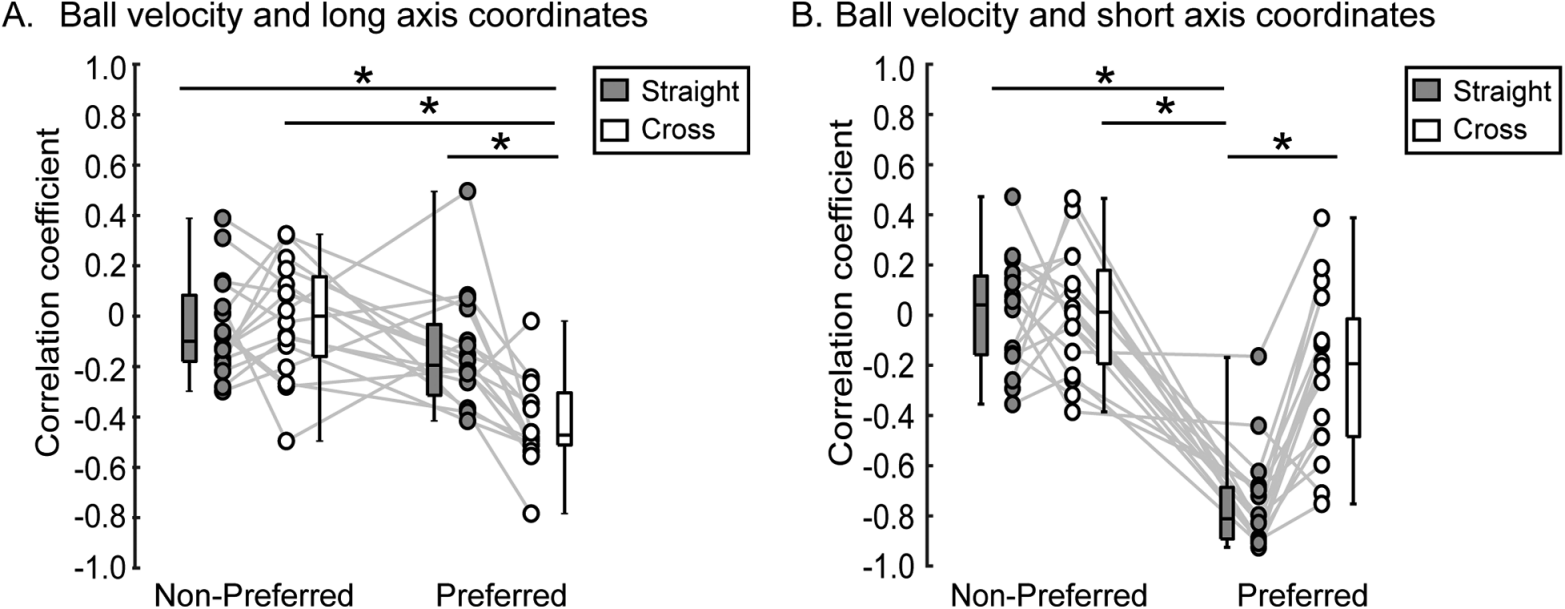
Correlation between ball long and short axis coordinates and ball velocity. The figure displays correlation between long axis of 95% equal confidence ellipse coordinates and ball velocity **(A)** and the correlation between short axis of 95% equal confidence ellipse coordinates and ball velocity **(B)**. The distribution of the data is depicted using box plots, showing minimum, maximum, median, and first and third quantiles. A significant difference between kicking foot and shoot direction was identified by post-hoc test (* in the figure, *p* < 0.05).

Different relationships between ball velocity and long or short axis coordinates of the ball position were observed for the kicking foot and shooting direction conditions (Figures 6A,B). An interaction between kicking foot and shooting direction conditions was observed in the correlation coefficients between ball velocity and the long [*F*(1, 15) = 19.53, *η*^2^ = 0.17, *p* < 0.001] and short [*F*(1.15) = 11.57, *η*^2^ = 0.11, *p* = 0.004] axis coordinates of the ball position. For the non-preferred foot condition, no significant correlations with ball velocity were observed for either the long- or short-axis coordinates of ball position (the 95% confidence interval between subjects for the correlation coefficients crossed zero). In the preferred foot shooting, for the straight condition, a correlation was observed where the ball velocity was higher for shots in the lower left long axis direction [*r* = −0.802, (−0.870, −0.704), 95% CI]. On the other hand, the correlation between shooting position in the short axis direction and ball velocity was *r* = −0.419 [−0.511, −0.318, 95% CI]. In the preferred foot shooting, in the cross condition, the correlation was observed that the lower right short-axis direction was associated with higher ball velocity.

## 6 Discussion

We initially examined the probabilistic structure of the ball positions for soccer shooting. The results showed an up-right ellipsoidal distribution for kicks with the right foot and an up-left distribution for kicks with the left foot. The orientation of the ellipse was not affected by the direction of the shot, whether straight or cross. We tested two hypotheses: that shooting precision would be higher for the preferred limb than for the non-preferred limb, and that it would be greater for the straight direction compared to the cross direction. The first hypothesis was confirmed by observing a smaller 95% confidence ellipse area, as well as shorter long and short axis lengths for the preferred foot compared to the non-preferred foot. This was also confirmed by the number of trials in which the kicked ball hit the target. The second hypothesis was rejected by the fact that there was no significant main effect of shoot direction nor significant foot*direction interaction.

In this study, we used two-dimensional normal distribution to evaluate the precision of the shooting. In Nagasawa et al. (8) and Radman et al. (29), soccer goal mouth was divided into 3 and 8 areas and scores were assigned for the areas for evaluation of kickers’ skill. In their method, the score was not proportional to the distance to the target, and an error was not quantified if the ball position was within the same area as the target. In addition, since the area outside the frame of a soccer goal was set to be zero, different score would be given depending on the direction of the error (e.g., if the target was set at the right top corner, a half score would be given for a leftward or downward errors, whereas a zero score would be given for a rightward or upward errors). Nagasawa et al. (8) reported that scores for the non-preferred foot were 1.17 times higher than those for the preferred foot. However, it does not quantitatively represent the variability of kicks from the motion control perspective (Figure 1A). In this mean, our finding (i.e., 1.7 times difference in the area of 95% equal confidence ellipse) was the first to quantify the difference in shot precision between the preferred and non-preferred feet in amateur soccer players.

According to the theory of the speed-accuracy tradeoff (17, 18), the observed difference in shooting precision could be explained by the difference in ball velocity if kicks by the preferred foot were slower than those by the non-preferred foot. Rakojević et al. (19) reported the principle applies to soccer kicking task in 13 junior soccer players. In our present study, we confirmed no significant differences between the preferred and non-preferred kicks were observed in the ball velocity during the tasks relative to the individual's maximum, indicating that the participants’ strategy of the relative weighting over speed and accuracy was consistent between the kicks by the preferred and non-preferred feet. Therefore, the observed difference in shooting precision should be attributed to the asymmetry in kicking skill between the preferred and non-preferred feet.

Motor control mechanisms explaining the observed difference in precision between kicks by preferred and non-preferred legs are open to discussion. We consider several aspects of motor control in the shooting task. Firstly, shooting is a complex task that includes various components. According to the review by Sainburg (30), laterality or limb dominance should be attributed to sensorimotor functions related to various components of motor tasks such as limb manipulation, impedance control, and stability control (30). A previous study focusing on lower limb motor control reported no difference in performance between single-legged balancing and vertical foot pointing tasks (31). In contrast, they observed significant asymmetry in CoP displacement during a combined foot pointing and balancing task. These results suggest that greater asymmetries may become evident when tasks are complex and include multiple functions. Another property of the shooting task that may enhance performance asymmetry is the short movement time. If the movement time is long enough to allow for feedback control of the limb, asymmetry in initial motor control could potentially be masked and not reflected in endpoint variability. It has been reported that the time required from backswing to follow-through in the soccer kicking motion is as short as 400 ms (32). This brief duration suggests that players must rely more heavily on feedforward control processes during shooting, which may accentuate inherent asymmetries between preferred and non-preferred legs.

There were no main effects of shooting direction or shooting direction*kicking foot interaction on the parameters of precision quantified in this study (95% equal confidence ellipse area, long axis, short axis, and long axis orientation). This does not support the hypothesis that shooting in the straight direction is more precise than shooting in the cross direction. In previous studies, Almeida et al. (3) analyzed 536 penalty kick attempts during 5 years of UEFA-sponsored matches and stated that the success rate of shooting in the straight direction was higher. Nagasawa et al. (8) also reported that shooting in the straight direction was more accurate than shooting in the cross direction. The present study showed the slope of the ellipse is up to the right if the shot is right-footed and down to the right if the shot is left-footed. In actual penalty kick and target shooting experiments, it is assumed that straight direction shots have a higher probability of being within the frame of the goal or hitting the target. The results seen in the previous studies are related to the fact that the variation is elliptical, and the accuracy of the shot may be the same regardless of the direction.

It should be noted that our findings were obtained from amateur players. The observed asymmetry in shooting accuracy between the preferred and non-preferred feet is in line with those reported in previous research (8). This discrepancy may be attributed to the disproportionate frequency of foot utilization in soccer games, particularly at amateur levels. Interestingly, professional soccer players have been shown to exhibit no significant disparity in performance success rates between their preferred and non-preferred feet, despite that the preferred foot is more frequently used than non-preferred foot in matches (4, 5). This suggests that attaining ambidexterity may be advantageous for ascending to professional levels. Marcori et al. (33) suggest that long-term practice improves the performance of the non-dominant hand. By extension, increased practice time with the non-preferred foot might lead to a smaller difference in performance between the preferred and non-preferred feet. Furthermore, the magnitude of the asymmetry in shooting precision may be a potential indicator of players’ skill level. Consequently, comparative analysis of shooting precision between preferred and non-preferred feet could yield valuable insights for player evaluation and development strategies.

Analyzing the determinants of ball position and its variability is of significant interest. According to Newtonian mechanics, the final ball position is governed by initial ball parameters: linear and angular velocity. Gordon et al. (12) postulated that the ellipsoidal distribution of arm reaching endpoints reflects independent parameters of motor control related to movement direction and amplitude. Consequently, we investigated the correlation between ball velocity and ball position along the long and short axes of the ball distribution. Theoretically, if the initial launch angle and spin parameters remain constant, balls with lower velocities would follow a more downward trajectory due to gravitational forces. However, our observations revealed counterintuitive results: in the straight condition with the preferred foot, faster balls tended to deviate downward and rightward along with the short axis; in the cross condition with the preferred foot, faster balls tended to deviate downward and leftward along with the long axis. These findings suggest the existence of a compensatory mechanism among ball parameters to ensure target acquisition. The discrepancy in correlational results—ball velocity correlating with ball position along the short axis for straight kicks and along the long axis for cross kicks—may be attributed to differences in ball spin characteristics between straight and cross kicks. Intriguingly, for the non-preferred foot, ball velocity did not correlate significantly with ball positions along either axis. This lack of correlation between ball velocity and final position implies that other ball parameters, such as launch angle and rotational properties, may vary without apparent coordination. While our limited measurements preclude a comprehensive explanation of the mechanisms underlying shot errors, it would be informative to employ more detailed biomechanical measurements to elucidate the factors influencing shot precision.

Several factors should be considered when interpreting the results of the present study. Firstly, to investigate the asymmetry in shooting precision, we used a paradigm where participants kick a stationary ball, similar to penalty kicks. This differs from real soccer games where players must use both feet depending on the situation. Secondly, our participants were able to pre-determine the shot direction according to the experimenter's instructions. It should be noted that different motor control mechanisms are involved when players are required to make real-time decisions under time pressure or uncertainty (34, 35). Finally, our results were obtained from amateur players and might differ from those of athletes at professional levels. Previous studies have reported an absence of significant asymmetry between the preferred and non-preferred feet in game statistics such as success rates of passes and shots among professionals. It would be interesting to investigate the differences in kick precision asymmetry under various psychological conditions and across different levels of expertise.

## Data Availability

Datasets are available upon request. The raw data supporting the conclusions of this article will be made available by the authors, without undue reservation.
